# Physical mapping of a new powdery mildew resistance locus from *Thinopyrum ponticum* chromosome 4AgS

**DOI:** 10.3389/fpls.2023.1131205

**Published:** 2023-02-23

**Authors:** Guotang Yang, Pingchuan Deng, Wanquan Ji, Shulan Fu, Hongwei Li, Bin Li, Zhensheng Li, Qi Zheng

**Affiliations:** ^1^ State Key Laboratory of Plant Cell and Chromosome Engineering, Institute of Genetics and Developmental Biology, The Innovative Academy of Seed Design, Chinese Academy of Sciences, Beijing, China; ^2^ College of Advanced Agriculture Sciences, University of Chinese Academy of Sciences, Beijing, China; ^3^ College of Agronomy, Northwest A & F University, Yangling, Shaanxi, China; ^4^ College of Agronomy, Sichuan Agricultural University, Chengdu, Sichuan, China

**Keywords:** *Thinopyrum ponticum*, 4Ag, *Pm* gene, cytogenetic analysis, specific marker amplification, physical mapping

## Abstract

*Thinopyrum ponticum* (Podp.) Barkworth and D.R. Dewey is a decaploid species that has served as an important genetic resource for improving wheat for the better part of a century. The wheat–*Th. ponticum* 4Ag (4D) disomic substitution line Blue 58, which was obtained following the distant hybridization between *Th. ponticum* and common wheat, has been stably resistant to powdery mildew under field conditions for more than 40 years. The transfer of 4Ag into the susceptible wheat cultivar Xiaoyan 81 resulted in powdery mildew resistance, indicating the alien chromosome includes the resistance locus. Irradiated Blue 58 pollen were used for the pollination of the recurrent parent Xiaoyan 81, which led to the development of four stable wheat–*Th. ponticum* 4Ag translocation lines with diverse alien chromosomal segments. The assessment of powdery mildew resistance showed that translocation line L1 was susceptible, but the other three translocation lines (WTT139, WTT146, and WTT323) were highly resistant. The alignment of 81 specific-locus amplified fragments to the *Th. elongatum* genome revealed that 4Ag originated from a group 4 chromosome. The corresponding physical positions of every 4Ag-derived fragment were determined according to a cytogenetic analysis, the amplification of specific markers, and a sequence alignment. Considering the results of the evaluation of disease resistance, the *Pm* locus was mapped to the 3.79–97.12 Mb region of the short arm of chromosome 4Ag. Because of its durability, this newly identified *Pm* locus from a group 4 chromosome of *Th. ponticum* may be important for breeding wheat varieties with broad-spectrum disease resistance.

## Introduction

*Blumeria graminis* f. sp. *tritici* (DC.) Speer (*Bgt*), which causes powdery mildew of wheat, is a biotrophic fungus that is distributed worldwide, but mainly in regions with dry and cool climates. In recent years, it has also spread to warmer and drier regions because of the intensive production of wheat at relatively high plant densities in irrigated fields in which nitrogen fertilizers are applied (Cowger et al., 2012; Singh et al., 2016). In China, *Bgt* races were initially isolated in the southwestern part of the country in the late 1970s, but they have since been detected in the eastern and northern regions (Xiao et al., 2022). Powdery mildew primarily damages wheat leaves and disrupts photosynthetic activities, ultimately leading to decreased plant growth and grain filling, with detrimental effects on wheat production (Zhang et al., 2020). From 2002 to 2020, powdery mildew annually affected an average of 7 million ha of the wheat-growing regions in China (Xiao et al., 2022). Hence, breeding disease-resistant wheat cultivars is critical for controlling powdery mildew.

To date, 65 permanently designated powdery mildew resistance genes (*Pm1*–*Pm68*) and dozens of provisionally named *Ml* genes have been identified in wheat and its relatives (McIntosh et al., 2021; Wu et al., 2021; Wu et al., 2022; Xiao et al., 2022). However, only a few resistance genes have been characterized *via* positional cloning and the application of emerging sequencing strategies. Most of these cloned *Pm* genes were derived from the secondary and tertiary gene pools of wheat, including *Pm8* from *Secale cereale* L. (2*n* = 2*x* = 14, RR) (Hurni et al., 2013), *Pm21* from *Haynaldia villosa* (L.) Schur [syn. *Dasypyrum villosum* (L.) Candargy, 2*n* = 2*x* = 14, VV] (He et al., 2018; Xing et al., 2018), *Pm41* from *Triticum turgidum* ssp. *dicoccoides* (2*n* = 4*x* = 28, AABB) (Li et al., 2020), and *Pm60* from *Triticum urartu* Thum. ex Gandilyan (2*n* = 2*x* = 14, AA) (Zou et al., 2018). These genes all encode nucleotide-binding leucine-rich repeat (NLR) proteins that recognize effectors and trigger the defense response to powdery mildew. Among these genes, *Pm8* and *Pm21* have been exploited for wheat breeding in China. Specifically, approximately 50%–60% of the varieties cultivated in major wheat-producing regions contain *Pm8* and more than 40 commercial varieties carry *Pm21* (Cao et al., 2015; Xing et al., 2021). Because of the rapid evolution of new *Bgt* isolates, some of the existing resistance genes can no longer protect plants from disease (Lu et al., 2020). For example, *Pm8*, which was derived from chromosome 1RS of Petkus rye, has no protective effect against the new *Bgt* isolate No. 9 collected in Sichuan, China (Ren et al., 2017). Similarly, the extensive and long-term application of *Pm21* has accelerated the evolution of *Bgt* races. Thus, *Pm21* will eventually become useless for controlling powdery mildew caused by new *Bgt* strains (He et al., 2020). Therefore, new resistance loci must be identified and applied in wheat breeding programs.

As one of the important wild relatives of wheat, tall wheatgrass [*Thinopyrum ponticum* (Podp.) Barkworth and D.R. Dewey, 2*n =* 10*x =* 70, E^e^E^e^E^b^E^b^E^x^E^x^StStStSt or JJJJJJJ^s^J^s^J^s^J^s^; syn*. Agropyron elongatum* (Host) P. Beauvois, *Lophopyron ponticum* (Podp.) Á Löve, *Elytrigia elongata* (Host) Nevski, *Elytrigia pontica* (Podp.) Holub.] is reportedly highly resistant to powdery mildew (Zhang et al., 1996b; Zhang et al., 1997; Chen et al., 1998; Li and Wang, 2009). However, only *Pm51* has been characterized. (Zhan et al., 2014). Briefly, *Pm51* in the wheat*–Th. ponticum* introgression line CH7086 was putatively revealed to be derived from *Th. ponticum* following an evaluation of seedling resistance (all stage resistance, ASR) and was mapped to wheat chromosome 2BL on the basis of a population linkage analysis. Additionally, some *Th. ponticum* chromosomes and small chromosomal segments have powdery mildew resistance-related loci. For example, the wheat–*Th. ponticum* 1J^s^ (1B) substitution line SN19647 and the 1J^s^ (1D) substitution line CH10A5 express *Pm* genes related to disease resistance (Wang et al., 2020b; Li et al., 2021). Although *Th. ponticum* 1J^s^ chromosomes are present in both lines, they were differentiated using molecular markers validation. Both substitution lines were proven to carry *Th. ponticum*-derived *Pm* genes by pedigree analysis at adult plant stage. The wheat–*Th. ponticum* translocation line 11-20-1 is immune to *Bgt* races at the seedling and adult plant stages (Li et al., 2017). A cytogenetic analysis detected a T5DL·5AgS translocation in this line. Furthermore, the alien segment was associated with powdery mildew resistance following the analysis of the F_2_ population derived from the cross between 11-20-1 and Yannong 19. Another wheat–*Th. ponticum* translocation line (WTT80), which carries a pair of TTh-1DS·1DL translocated chromosomes, is immune to *Bgt* isolate E09 at the seedling stage. The resistance gene(s) came from *Th. ponticum* according to a pedigree analysis (Yang et al., 2022). The wheat–*Th. ponticum* introgression lines SN0293-2 and SN0293-7 exhibit broad-spectrum powdery mildew resistance at the seedling and adult plant stages. Field tests of the parents suggested the *Pm* gene in these two lines was likely from *Th. ponticum* (Li et al., 2022). Because *Th. ponticum* is a decaploid species with a complex and large genome, additional *Pm* genes will need to be identified and functionally annotated in future studies.

The blue-grained wheat line (Blue 58), was derived from the distant hybridization between common wheat variety Dami 953 and *Th. ponticum*, followed by successive backcrossing to wheat cultivars 573, Aifeng 3 and Tianxuan 15, during which wheat chromosome 4D was substituted by *Th. ponticum* chromosome 4Ag (Mu et al., 1986). The blue grain phenotype is caused by the accumulation of anthocyanin pigments in the aleurone layer. Wheat geneticists and breeders have used the blue grain-related gene as a marker to calculate the frequency of natural crosses, identify true hybrids, and monitor chromosomal changes (Zeven, 1991; Hucl and Matus-Cadiz, 2001; Metzger and Sebesta, 2004; Morrison et al., 2004; Zheng et al., 2006; Shen et al., 2013; Liu et al., 2018a). To determine the physical position of the gene responsible for the blue aleurone, a series of translocation lines was developed *via* irradiation. The blue grain-related gene was physically mapped to regions [0.71–0.80 and 0.75–0.89 relative fragment lengths (FL)] on the long arm of chromosome 4Ag (Zheng et al., 2006; Liu et al., 2018a). A cytogenetic analysis revealed that 4Ag was a recombinant chromosome. Its centromeric and pericentromeric regions were from the E-genome, whereas the distal regions of the two arms were from the St-genome (Zheng et al., 2006). In this study, we confirmed that chromosome 4Ag from Blue 58 also has a locus conferring excellent resistance to *Bgt* races at the adult plant stage. Furthermore, we constructed a relatively refined physical map of chromosome 4AgS and mapped the *Pm* locus using a series of translocation lines. The results of this study provide important insights into the *Th. ponticum* group 4 chromosome*-*derived *Pm* gene conferring adult plant resistance (APR). Moreover, the new translocations may be useful for wheat breeding programs.

## Materials and methods

### Plant materials

**Figure 1:**
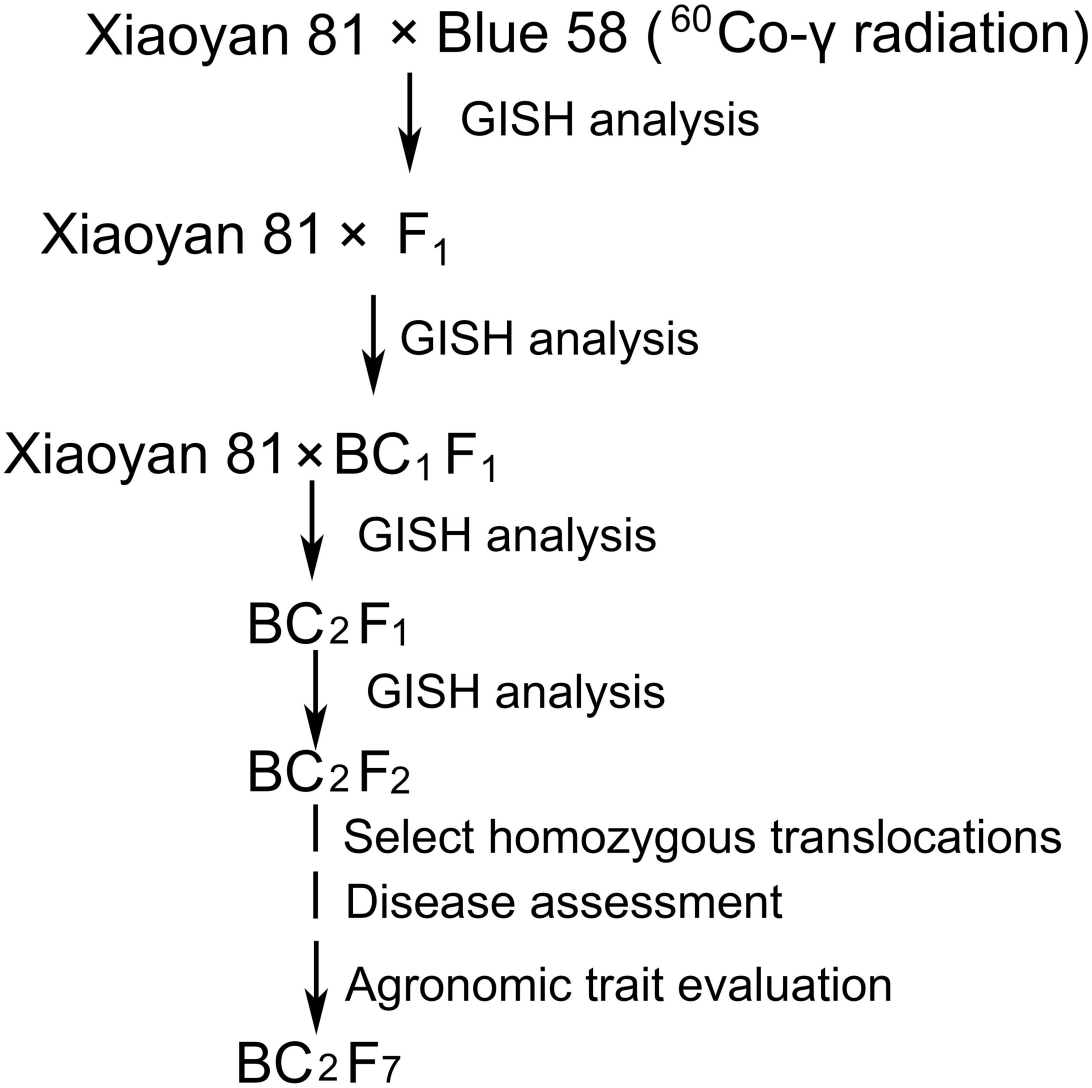
Procedure for producing wheat**–***Th. ponticum* 4Ag translocation lines.

Four wheat**–** *Th. ponticum* 4Ag translocation lines (L1, WTT139, WTT146, and WTT323) were developed from the cross Xiaoyan 81*2//Blue 58/Xiaoyan 81 (**Figure 1**). Firstly, wheat cultivar Xiaoyan 81 was pollinated with the ^60^Co γ-ray-irradiated pollen of Blue 58. Then, selected translocation lines were backcrossed twice with the recurrent parent Xiaoyan 81. Finally, stable translocation lines were obtained from the self-bred seeds. One wheat**–** *Th. ponticum* 4Ag substitution line 19ZQ43 was developed from the same cross without irradiation. To further confirm the genetic locus and accelerate its utilization in wheat improvement, the alien segment of WTT146 was transferred into widely adaptable cultivar Jimai 22 and a new stable translocation line J146 was developed. Xiaoyan 81, a high quality wheat cultivar, was developed from the cross Xiaoyan 54/8602 and approved for release by the Crop Breeding Examination Committee of Hebei Province (China) in 2005 (No. 2005006). Wheat cultivars Xiaoyan 81, Jimai 22, Xuezao and Chinese Spring (CS) are all preserved at the laboratory of Zhensheng Li, Institute of Genetics and Developmental Biology (IGDB), The Innovative Academy of Seed Design, Chinese Academy of Sciences.

### Cytogenetic identification

Chromosome preparation was carried out according to Han et al. (2006, 2009) with minor modifications. In brief, the seed roots with the length of 1-2 cm were collected, pretreated with N_2_O under 10 atm pressure for 2 hours and then fixed in 90% acetic acid for 8 min. The root tips were cut and digested with 2% cellulase and 1% pectinase before washed with 75% ethyl alcohol. The root tips were mashed using dissecting needle and diluted with 100% acetic acid. Finally, 10 μL mixture was dropped onto the center of a slide. The slides with good mitotic phases were used in sequential genomic *in situ* hybridization (GISH) and multi-color fluorescence *in situ* hybridization (mc-FISH).

The genomic DNA (gDNA) of *Th. ponticum* was labeled as a probe with Alexa Fluor-488-dUTP (green). While the gDNA of CS was used as a block. The probe and block with ratio of 1:200 were used in GISH analysis. After GISH, two repetitive probes, pAs1 (GenBank Accession number: D30736.1), isolated from *Aegilops tauschii* Coss. and labeled with Texas-red-5-dCTP (red), pSc119.2 (GenBank Accession number: KF719093), isolated from *S. cereale* and labeled with Alexa Fluor-488-dUTP (green), were used in the FISH analysis (Rayburn and Gill, 1986; McIntyre et al., 1990). After hybridization, the slides were washed in 2× Saline Sodium Citrate buffer followed by counterstain with 4, 6-diamidino-2-phenylindole (DAPI). The cells with clear hybridization signals were photographed by a DP80 CCD camera attached to an Olympus BX53 and analyzed using the program CellSens Standard 1.12 (Olympus, Tokyo, Japan).

### Powdery mildew resistance evaluation

The *Pm* resistance of five wheat**–** *Th. ponticum* translocation lines (L1, WTT139, WTT146, WTT323, and J146), one substitution line 19ZQ43 and their parents (Blue 58, Xiaoyan 81, and Jimai 22) were evaluated at the booting and filling stages. Plants were grown in two consecutive seasons (2020–2021 and 2021–2022) at Changping Experiment Station, IGDB, Beijing, China and one growing season (2021–2022) in Wenjiang, Chengdu, Sichuan, China. Wheat cultivar XueZao was planted next to the test rows as an inoculum spreader. The materials were naturally infected by powdery mildew. The infection types (ITs) were scored on a 0-4 scale according to the described by Fu et al. (2014), where 0-2 represent resistance and 3-4 represent susceptible.

### Sequence alignment and molecular marker analysis

In a previous study, gDNA extracted from Blue 58, CS, and *Th. ponticum* was digested using *Hae*III (New England Biolabs, America) and then specific fragments (400–450 bp) were selected for the specific-locus amplified fragment sequencing (SLAF-seq) analysis. The SLAFs of 4Ag were obtained following a comparison with the A-genome, D-genome, and the SLAFs of CS and *Th. ponticum*. Our group developed 573 markers specific to chromosome 4Ag on the basis of these SLAFs of 4Ag (Liu et al., 2018a), of which 223 markers specific to chromosome 4AgS were used for the subsequent analysis. First, the SLAFs related to these markers were aligned with the *Thinopyrum elongatum* (Host) D.R. Dewey (2*n* = 2*x* = 14, EE) genome (Wang et al., 2020a) to reveal the homoeologous group that 4Ag belongs to and with the *Th. ponticum* genome (unpublished) to identify specific chromosome. The comparisons were completed using Bowtie2, with one mismatch accepted. The correct sequence was obtained if the physical distance between the forward and reverse SLAFs was less than 500 bp. These SLAFs were further mapped to a specific chromosome to determine their physical positions using BLAST, with an E-value cut off of 1e-5. Depending on the physical positions, some markers were selected and amplified by PCR using the Blue 58 gDNA. The amplified products were sequenced and aligned with the *Th. ponticum* and *Th. elongatum* genomes. Syntenic relationships were visualized using MapChart 2.32 (Voorrips, 2002). Finally, the chromosomal regions of four wheat–*Th. ponticum* translocation lines (L1, WTT139, WTT146, and WTT323) were determined according to a PCR amplification of these selected markers. The PCR amplification and detection were performed as described by Liu et al. (2018a).

### Agronomic traits evaluation

In 2018–2019 and 2019–2020, the recurrent parent Xiaoyan 81 and the translocation line WTT146 were grown in triplicate on plots at Nanpi Eco-Agricultural Experimental Station, Chinese Academy of Sciences, Hebei Province, China (116.4°E, 38.0°N). The soil salinity of this station was 0.18% (m/m) according to the detection values in decades. In 2020–2021 and 2021–2022, the recurrent parent Xiaoyan 81 and the translocation lines L1, WTT139 and WTT323, the recurrent parent Jimai 22 and the translocation line J146 were grown in triplicate on plots at Changping Experiment Station, IGDB, Beijing, China (116.2°E, 40.6°N). A total of 20 seeds were sown in a 2.0 m row with an inter-row spacing of 0.2 m. Field managements were carried out according to the common practices for wheat production with no irrigation at Nanpi station and adequate irrigation at Changping station during the whole growing seasons. Five plants located in the center of middle row were selected and investigated for agronomic traits at physiological maturity. The agronomic traits mainly included plant height (PH), effective tiller number (ETN), kernel number per main spike (KNMS), total kernel number (TKN), thousand-kernel weight (TKW) and yield per plant (YPP). The mean value and standard deviation were calculated using SPSS version 19.0 software (IBM, New York, USA). Significant difference was determined by the Student’s *t* test. One-way ANOVA analysis was conducted using data processing system (Tang and Zhang, 2013).

## Results

### Powdery mildew APR locus from *Th. ponticum* chromosome 4Ag

**Figure 2:**
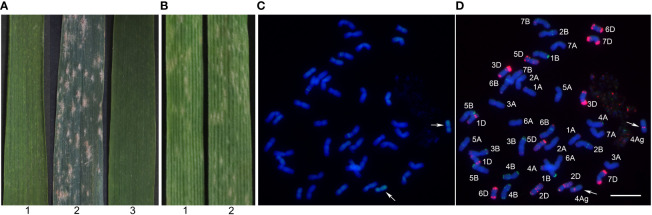
The powdery mildew resistance evaluation and cytogenetic analysis. **(A)** The powdery mildew resistance evaluation at the adult plant stage. **(B)** The powdery mildew resistance evaluation at the seedling stage. **(C)** The GISH pattern of 19ZQ43 probed with total genomic DNA of *Th. ponticum*. **(D)** The mc-FISH pattern of 19ZQ43 using probes pAs1 (red) and pSc119.2 (green). 1: Blue 58; 2: Xiaoyan 81; 3: 19ZQ43. The arrows note a pair of 4Ag chromosomes. Bar = 20 μm.

At the bolting and filling stages, Blue 58 was highly resistant to powdery mildew (IT = 1), whereas Xiaoyan 81 was susceptible to powdery mildew (IT = 4) (**Figure 2A**). At the seedling stage, Blue 58 and Xiaoyan 81 were inoculated with *Bgt* race E09 and the resulting IT scores were 3 and 4, respectively (**Figure 2B**), suggesting Blue 58 carried the *Pm* gene responsible for APR. To determine the origin of the resistance locus, we transferred 4Ag into the powdery mildew-susceptible cultivar Xiaoyan 81 and produced a new stable substitution line (19ZQ43) with the pedigree Xiaoyan 81*3/Blue 58. The 19ZQ43 grains were blue, similar to the Blue 58 grains. The GISH analysis indicated that 19ZQ43 had 42 chromosomes, including 40 wheat chromosomes and two alien chromosomes (**Figure 2C**). The mc-FISH analysis revealed a strong pAs1 signal at the pericentromeric region of the short arm of the alien chromosomes, implying that 19ZQ43 carried a pair of 4Ag chromosomes (**Figure 2D**). The evaluation of resistance showed that 19ZQ43 was resistant to powdery mildew at the adult plant stage (IT = 1) (**Figure 2A**). Thus, we speculated that *Th. ponticum* chromosome 4Ag contains an adult-stage powdery mildew resistance locus.

### Development, cytogenetic identification, and disease resistance evaluation of novel translocation lines

**Figure 3:**
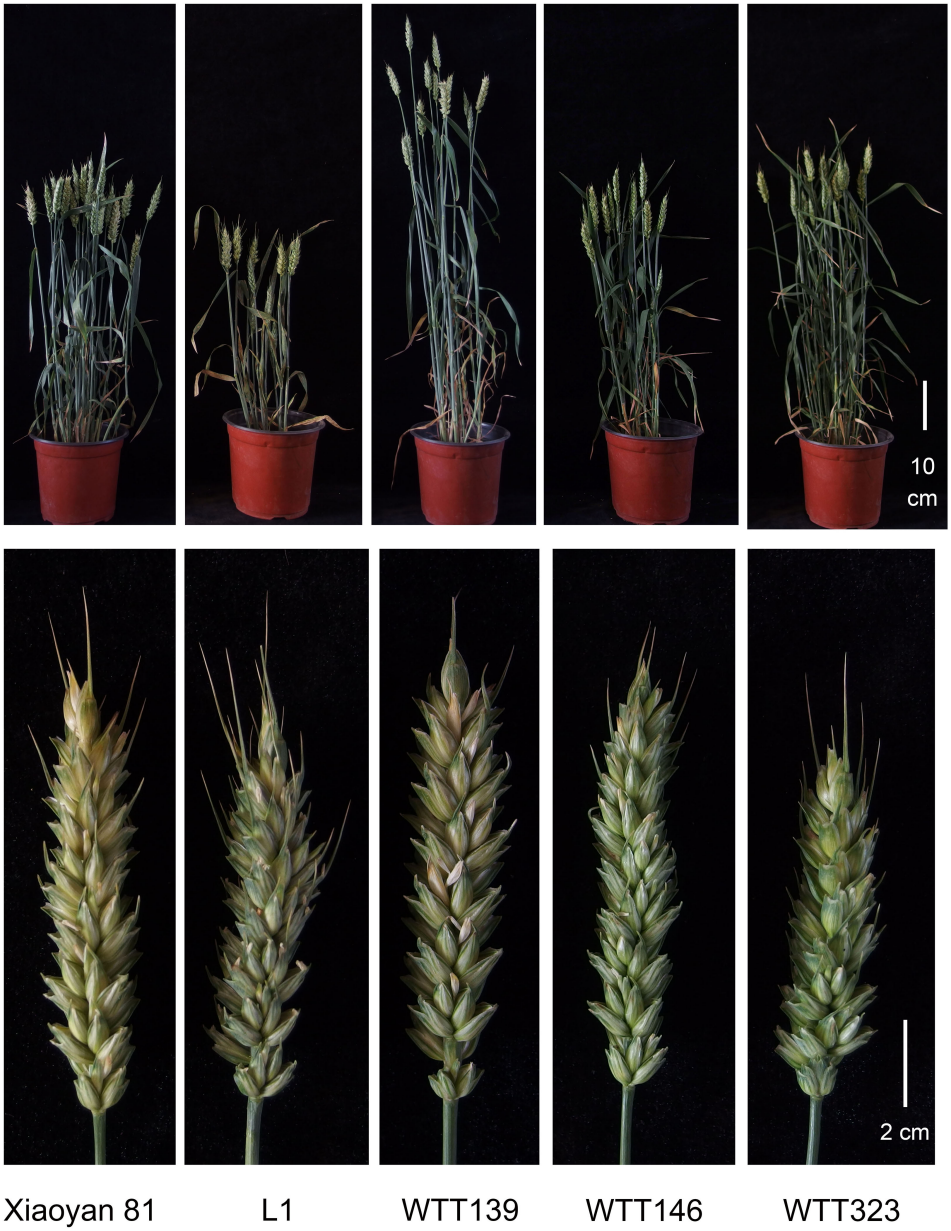
Phenotypic images of Xiaoyan 81 and four wheat**–***Th. ponticum* 4Ag translocation lines.

**Figure 4:**
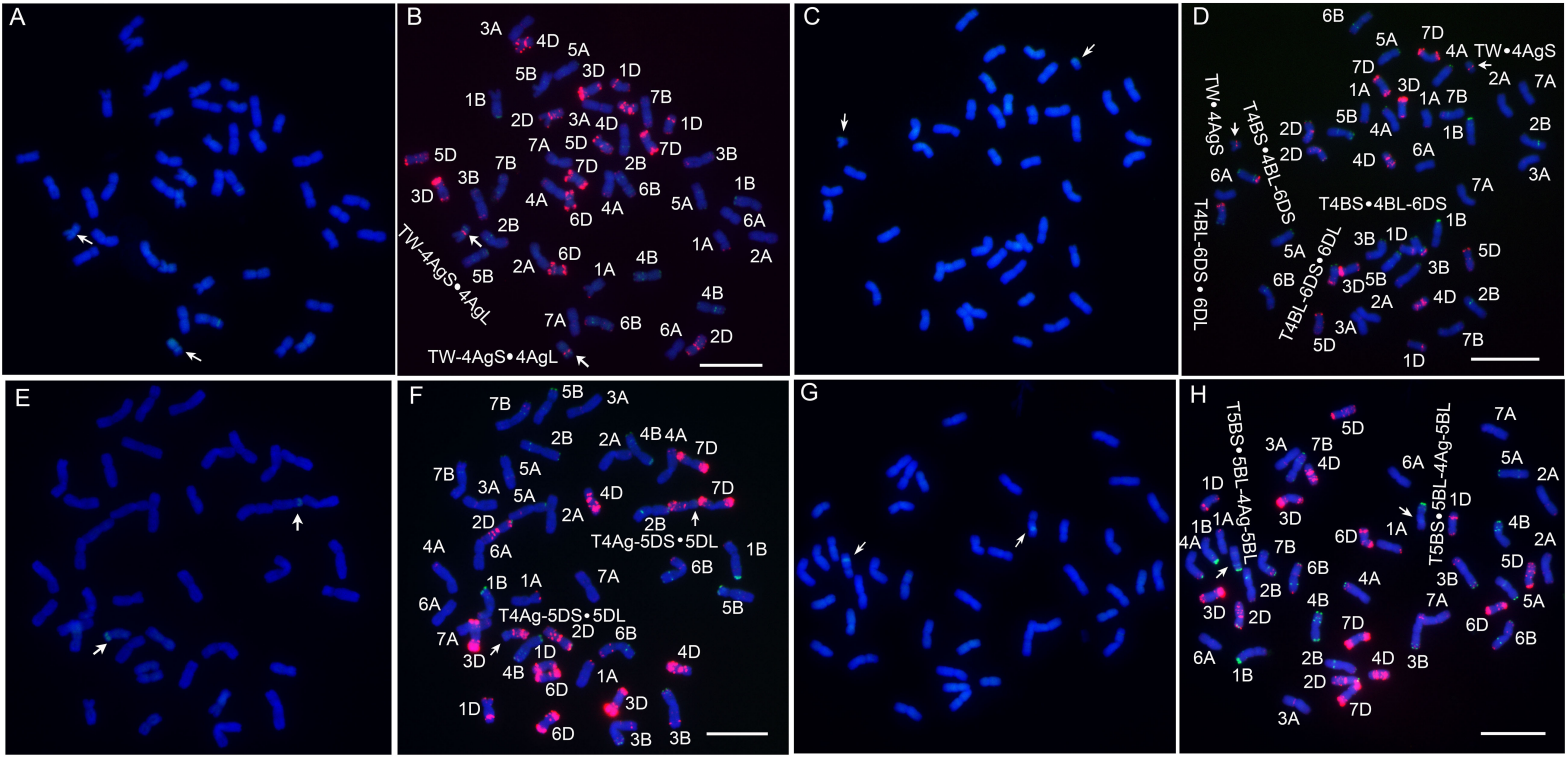
Sequential GISH and mc-FISH analysis of translocation lines. **(A)** The GISH pattern of L1 with total genomic DNA of *Th. ponticum* as a probe. **(B)** The mc-FISH pattern of L1 using two probes pAs1 (red) and pSc119.2 (green). **(C)** The GISH pattern of WTT139. **(D)** The mc-FISH pattern of WTT139. **(E)** The GISH pattern of WTT146. **(F)** The mc-FISH pattern of WTT146. **(G)** The GISH pattern of WTT323. **(H)** The mc-FISH pattern of WTT323. The arrows note a pair of translocated chromosomes. Bar = 20 μm.

Four novel translocation lines were developed *via* irradiation and their chromosomal compositions were characterized (**Figure 3**). Among them, L1 has blue seeds, while lines WTT139, WTT146 and WTT323 have white seeds. The GISH analysis showed that L1 had 44 chromosomes, of which 42 were detected as blue chromosomes and two were detected as chromosomes with small blue distal ends and large green chromosomal segments (**Figure 4A**). Hence, L1 likely contained 21 pairs of complete wheat chromosomes and one pair of wheat–*Th. ponticum* translocated chromosomes with large alien fragments. The mc-FISH analysis of the translocated chromosome of L1 detected a strong pAs1 signal near the centromere zone and a clear pSc119.2 signal near the terminal region of the short arm (**Figure 4B**), indicating that the intercalary region of 4AgS was broken and the larger alien chromosomal segment was fused to an unknown wheat chromosomal segment to produce the novel translocated chromosome TW-4AgS·4AgL. A total of 44 chromosomes were identified in WTT139 (**Figure 4C**). The mc-FISH analysis detected an obvious pAs1 band in the pericentromeric region of the alien chromosomal segment (**Figure 4D**), suggesting that 4AgS was translocated to a small unknown wheat chromosomal fragment to produce the new translocated chromosome TW·4AgS. In addition, reciprocal translocations occurred between chromosomes 4B and 6D. The GISH results for WTT146 revealed 40 chromosomes with blue signals and two blue chromosomes with green signals at the distal ends of their short arms (**Figure 4E**), implying that WTT146 was affected by the translocation of a small alien fragment. The mc-FISH analysis of the translocated chromosome detected clear pAs1 signals in the pericentromeric, intercalary, and terminal regions of the long arm, which was consistent with the signal pattern of chromosome 5DL (**Figure 4F**). The lack of an obvious signal prevented the accurate determination of the location of the alien segment in 4Ag. Thus, a T4Ag-5DS·5DL translocation event likely occurred in WTT146. The GISH analysis of WTT323 detected 40 chromosomes with blue signals and two chromosomes with blue signals as well as small regions with green signals (**Figure 4G**), indicating it had 20 pairs of intact wheat chromosomes and a pair of intercalary wheat**–** *Th. ponticum* translocated chromosomes. The mc-FISH analysis detected the typical pSc119.2 bands of chromosome 5B in the terminal and intercalary regions of the short arm of the translocated chromosome (**Figure 4H**), suggesting a small unknown alien segment was inserted into wheat chromosome 5B, resulting in the translocated chromosome T5BS·5BL-4Ag-5BL.

**Figure 5:**
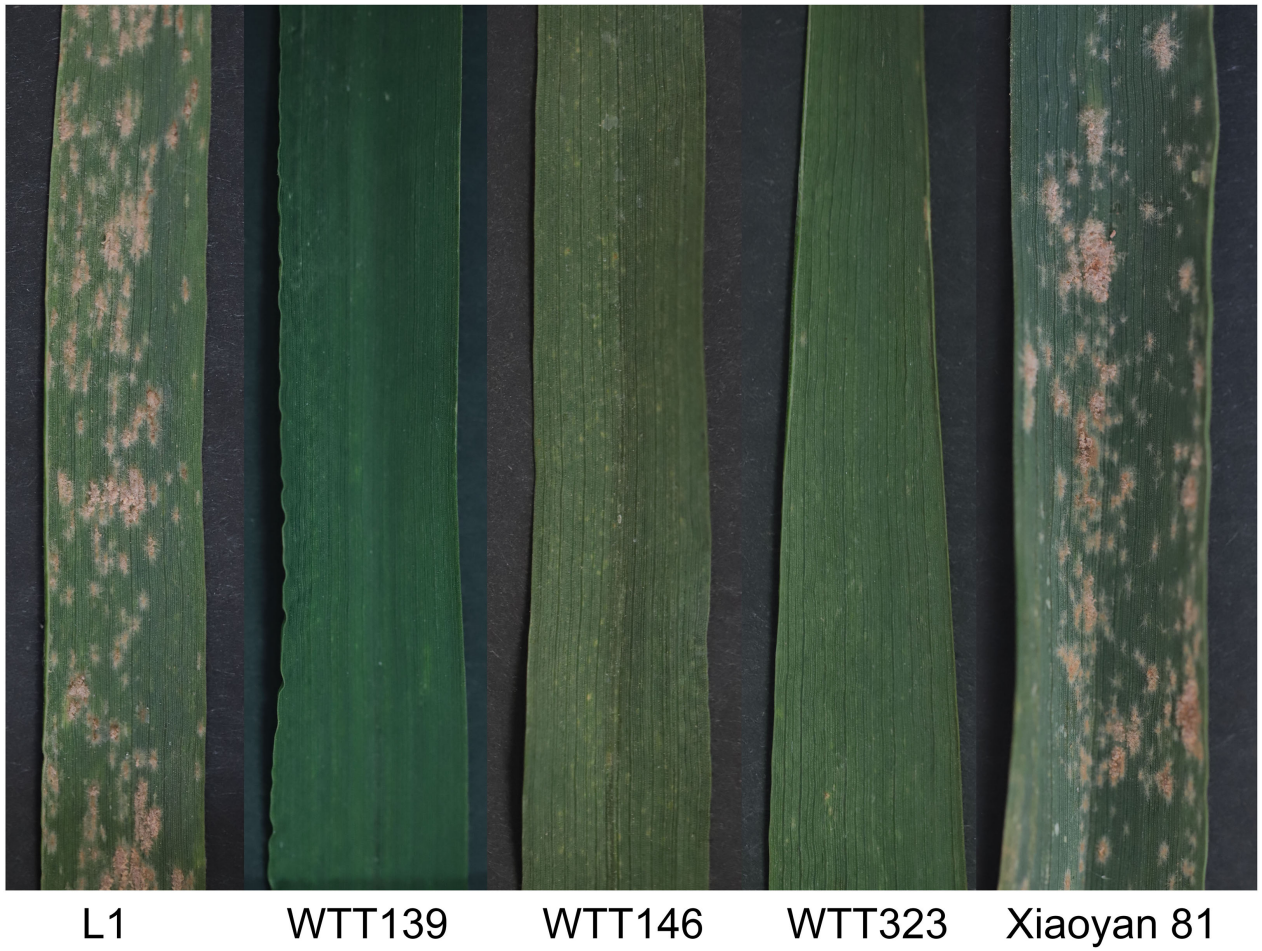
The powdery mildew resistance responses of four translocation lines and Xiaoyan 81.

At the adult plant stage, consistent IT scores were obtained in Beijing and Sichuan. Specifically, the IT scores were recorded when the powdery mildew spores fully covered the leaves of the susceptible control Xiaoyan 81. Among the materials that were evaluated in terms of their disease symptoms, WTT139, WTT146, and WTT323 were highly resistant to powdery mildew (IT = 1), similar to their resistant parent Blue 58, whereas L1 and Xiaoyan 81 were highly susceptible (IT = 4) (**Figure 5**; **Table 1**).

**Table 1:** Powdery mildew resistance assessment of Blue 58, its derived lines, Xiaoyan 81 and Jimai 22.

Years and sites	Materials	ITs	Number of plants
2020-2021 2021-2022 Beijing	Blue 58	1	60
	19ZQ43	1	60
	L1	4	60
	WTT139	1	60
	WTT146	1	60
	WTT323	1	60
	Xiaoyan 81	4	60
	J146	1	60
	Jimai 22	3	60
2021-2022 Sichuan	Blue 58	1	10
	L1	4	10
	WTT139	1	10
	WTT146	1	10
	WTT323	1	10
	Xiaoyan 81	4	10

### Alignment of selected 4AgS SLAF sequences

**Figure 6:**
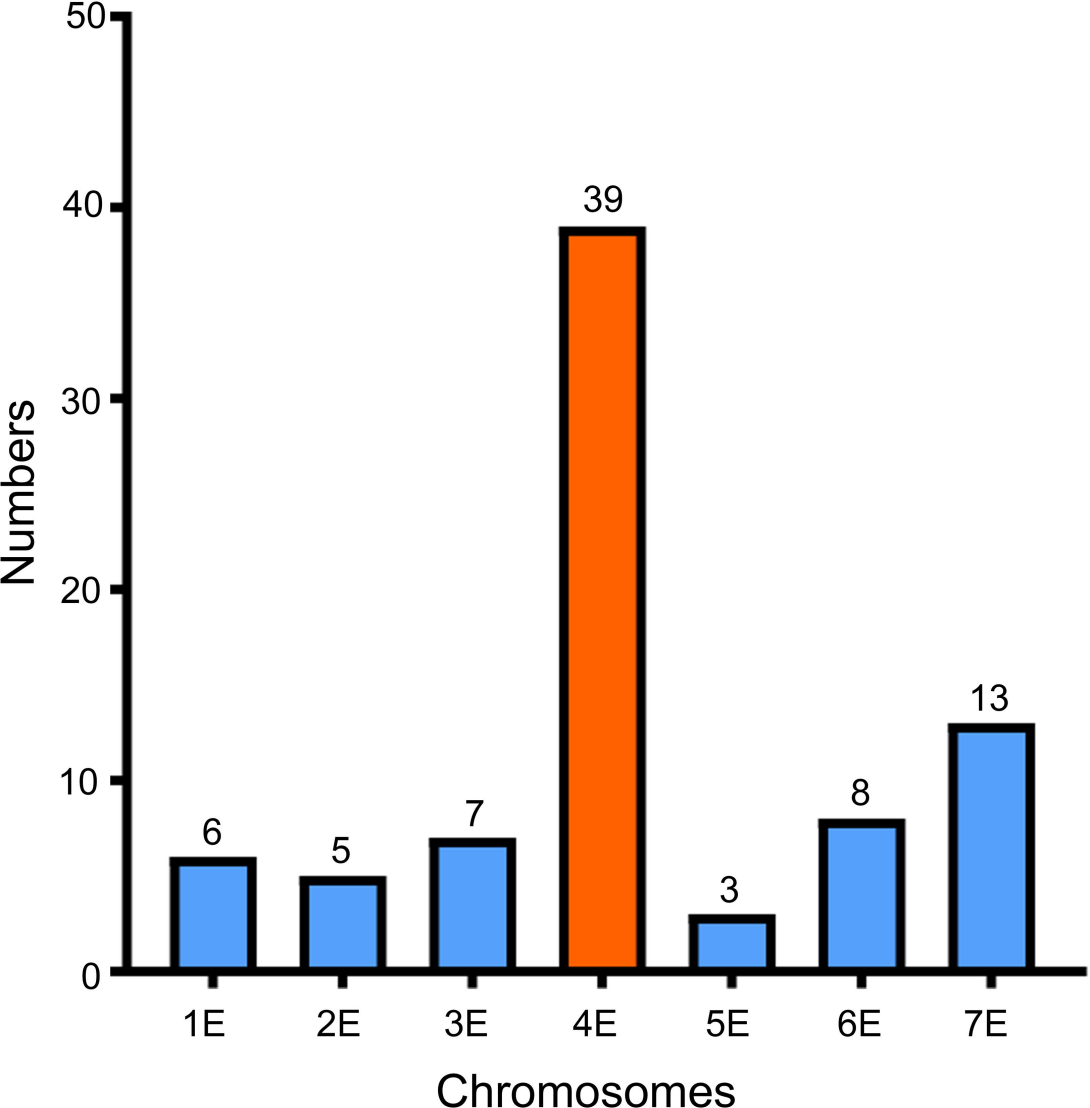
Alignment results of 81 specific SLAFs to *Th. elongatum* genome using Bowtie 2.

Because the powdery mildew-resistant line WTT139 only carries 4AgS, whereas the susceptible line L1 lacks part of a 4AgS segment, we speculated that the *Pm* locus was derived from 4AgS. In our previous study, 223 markers specific to chromosome 4AgS have been developed using SLAF-seq technology (Liu et al., 2018a). To further determine which homoeologous group 4Ag belongs to, the SLAFs related to these markers were compared with the *Th. elongatum* genome sequence using Bowtie2. On the basis of the sequence alignment, 81 of the 223 SLAFs were mapped to the *Th. elongatum* genome, and 39 of them (48.15%; largest proportion) were located on chromosome 4E (**Figure 6**; **Supplementary Table 1**). Therefore, 4Ag was proved to belong to homoeologous group 4 of *Th. ponticum*.

These 223 SLAFs were also compared with the *Th. ponticum* genome (unpublished) using the same parameters. The alignment results showed that 78 of the 223 SLAFs could be mapped to the *Th. ponticum* genome and 34 of these 78 SLAFs (43.59%; largest proportion) were located on chromosome LG34 (temporary name) (**Supplementary Table 2**). Thus, we identified chromosome 4Ag as chromosome LG34.

### Physical mapping of the *Pm* locus on chromosome 4AgS

**Figure 7:**
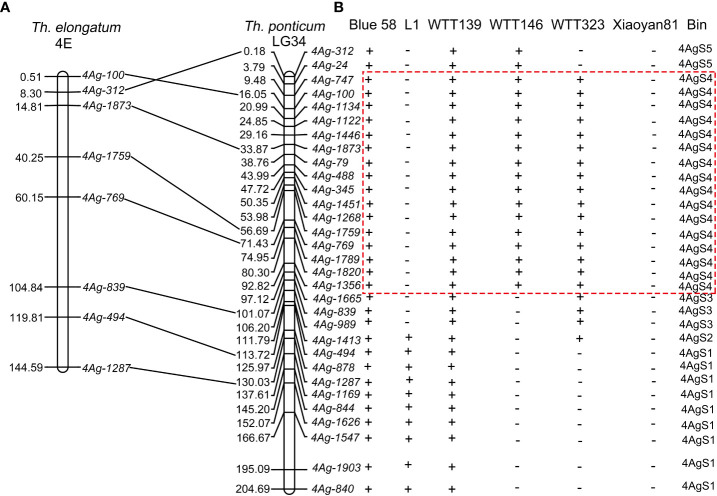
Synteny analysis and PCR amplification. **(A)** Synteny relationship of specific marker amplification products between *Th. elongatum* chromosome 4E and *Th. ponticum* chromosome LG34. **(B)** Amplified results in Blue 58, four translocation lines and Xiaoyan 81. The symbols ‘+’ and ‘−’ indicate the presence and absence of the specific marker loci, respectively. The dotted box indicates the physical region of *Pm* locus.

To elucidate the physical positions of the 223 specific markers, their SLAFs were mapped to chromosome LG34 using the BLAST online tool. A total of 113 SLAFs were mapped to chromosome LG34. More specifically, the forward and reverse SLAF sequences were highly similar (>90%) to LG34 sequences and SLAF sequences were separated by fewer than 500 bp (**Supplementary Table 3**). According to the BLAST sequence alignment results, 31 markers were selected for the PCR amplification of the Blue 58 gDNA (i.e., one marker per 5 Mb). The 31 PCR products were sequenced and then compared with the chromosome LG34 sequence. The physical positions of the 31 PCR products were close to the physical positions of their SLAFs (**Supplementary Table 4**). Additionally, eight of the 31 PCR products were similar to sequences on *Th. elongatum* chromosome 4E (sequence identities >85%) and revealed the good collinearity between *Th. elongatum* chromosome 4E and *Th. ponticum* chromosome LG34 (**Figure 7A**; **Supplementary Table 4**). The primers for these 31 specific markers were used to amplify the gDNA of Blue 58, L1, WTT139, WTT146, WTT323, and Xiaoyan 81. These 31 markers were assigned to the following five bins according to the amplification results: 4AgS1, 4AgS2, 4AgS3, 4AgS4, and 4AgS5, which contained 9, 1, 3, 16, and 2 markers, respectively (**Figure 7B**). The four translocation lines included different 4AgS chromosomal bins. Specifically, WTT139 contained all five chromosomal bins, with 0–204.69 Mb on chromosome LG34; L1 included bins 4AgS1–2 (106.20–204.69 Mb); WTT146 contained bins 4AgS4–5 (0–97.12 Mb); and WTT323 included bins 4AgS2–4 (3.79–113.72 Mb). After analyzing the IT scores and the alien segments in the four translocation lines, the *Pm* locus was mapped to bin 4AgS4. According to the physical positions of these specific markers, the *Pm* locus was narrowed to the 3.79–97.12 Mb region on 4AgS.

### Genetic analysis of the powdery mildew resistance locus

**Figure 8:**
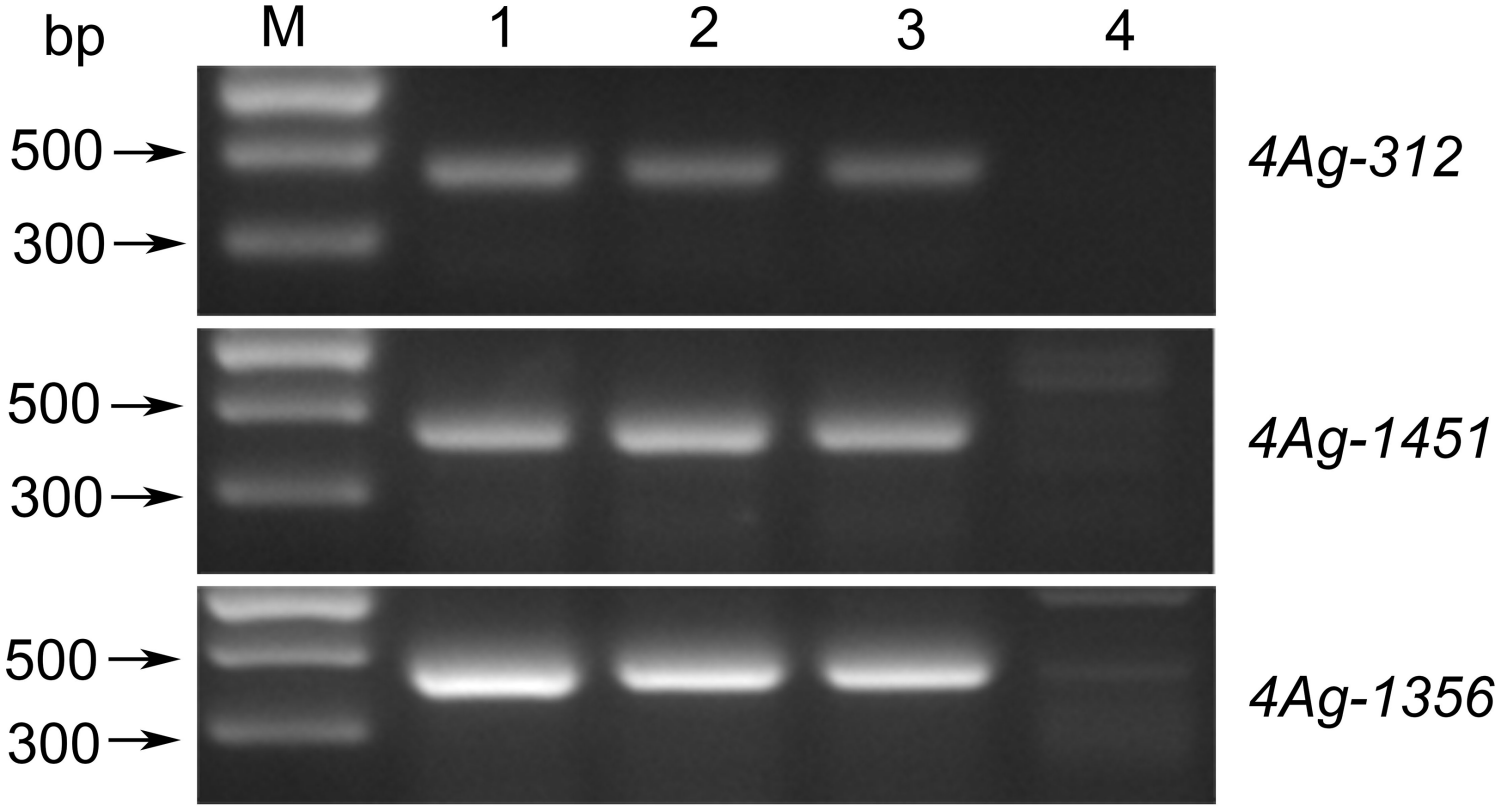
Amplified results of three specific markers of WTT146. M: Marker II; 1: Blue 58; 2: WTT146; 3: J146; 4: Jimai 22.

To further determine the genetic effect of the *Pm* locus on 4AgS, we transferred the alien segment of WTT146 into wheat cultivar Jimai 22 to produce the novel stable translocation line J146, which was verified by the amplification of specific markers (**Figure 8**). The evaluation of powdery mildew resistance showed that the recurrent parent Jimai 22 was susceptible to *Bgt* races (IT = 3), but J146 was highly resistant (IT = 1) (**Table 1**). Therefore, the *Pm* locus of WTT146 was probably transferred into J146. These findings provided additional evidence that this *Pm* locus contributes to powdery mildew resistance.

The agronomic traits of L1, WTT139, WTT146 and WTT323 and its recurrent parent Xiaoyan 81 were investigated during the 2018–2022 growing seasons (**Supplementary Table 5**). Unfortunately, the yields of these lines were significantly lower than the recurrent parent Xiaoyan 81 except for WTT139. In order to improve their yield traits, we have transferred their alien chromosomal segments into wheat cultivar Jimai 22. The novel translocation line J146 was thus developed. The agronomic traits of J146 and its recurrent parent Jimai 22 were investigated during the 2021–2022 growing seasons (**Supplementary Table 6**). There were no significant differences in PH, ETN, KNMS, and YPP. In contrast, compared with Jimai 22, J146 had a significantly lower TKW (*P* < 0.01), but a significantly higher TKN (*P* < 0.01). In other words, there was no obvious linkage drag associated with the alien segment of J146, making it potentially useful for increasing the powdery mildew resistance in wheat breeding.

## Discussion

Tall wheatgrass, which is one of the most important wild relatives of wheat, has been extensively used for the genetic improvement of wheat. To date, many elite cultivars derived from the distant hybridization between wheat and *Th. ponticum* have been approved and widely cultivated in China. For example, the high-quality and multi-resistant cultivar Xiaoyan 6 received the first prize for the National Award for Invention in 1985 (Hao et al., 2020). More than 60 cultivars have been derived from this backbone parent (Ma et al., 2018). Moreover, many essential resources, including wheat**–** *Th. ponticum* partial amphiploids [e.g. Xiaoyan series (Group of remote hybridization, 1977; Zhang et al., 1996a; Zheng et al., 2015), Agrotana (Chen et al., 1995), PWM series (Fedak et al., 2000), SS series (Oliver et al., 2006), BE-1 (Sepsi et al., 2008) and SN series (He et al., 2013; He et al., 2017; Pei et al., 2018)], addition lines [e.g. Ji791 and Ji924 (Li et al., 2003), W210, W211 and W212 (Wang and Wang, 2016), Xiaoyan 85 (Li et al., 2016) and WTA series (Jia et al., 2022)], substitution lines CH1113-B13 (Zhu et al., 2017), translocation lines [e.g. KS10-2 and KS24-1 (Kim et al., 1993; Friebe et al., 1996), RWG33 and RWG34 (Niu et al., 2014), SDAU1881 and SDAU1886 (Guo et al., 2015), Xiaoyan 447 (Li et al., 2016), Xiaoyan 851 (Li et al., 2019), WTT34 (Yang et al., 2021)] and introgression lines Shanrong 3 (Xia et al., 2003), have been developed *via* distant hybridizations and chromosome engineering. These cultivars and engineered materials have facilitated the transfer of valuable genes from *Th. ponticum* to wheat.

Considering the restricted recombination between alien chromatin and wheat homoeologous sequences, it is difficult to narrow down the regions containing these valuable foreign genes using regular genetic techniques. Translocations due to the irradiation of pollen or the *ph1b* mutant that divide chromosomal regions may be used to map foreign genes. For example, in our previous study (Liu et al., 2018a), we used eight wheat–*Th. ponticum* 4Ag translocation lines to locate the gene responsible for the blue grain phenotype. Lin et al. (2022) used four deletion lines and two translocation lines to map the powdery mildew resistance locus in the 6PL bin (0.27–0.51) of *Agropyron cristatum* (L.) Gaertn. (2*n* = 4*x* = 28, PPPP). In addition, because the CS *ph1b* mutant can significantly increase meiotic homoeologous recombination, it has been widely used to induce translocations. Men et al. (2022) developed 12 CS–*Ae. biuncialis* 2M^b^ recombinants through *ph1b*-induced homoeologous recombination. By combining the results of a cytogenetic analysis and an evaluation of the powdery mildew resistance of these recombinants, *Pm2Mb* was mapped to the 2M^b^L bin (0.49–0.66). For species lacking sequenced genomes, their chromosomes were not precisely characterized and only relative FLs were defined when we physically mapped a valuable gene. However, specific physical positions were determined for species with sequenced genomes. Thus, sequenced genomes accelerated the physical mapping of valuable genes, enabling them to be exploited in breeding programs. Fortunately, the genomes of *Th. elongatum* (Wang et al., 2020a) and *Th. ponticum* (unpublished) have been sequenced and assembled, in part because of the advances in the whole-genome sequencing of wild relatives of wheat. Thus, we were able to map the *Pm* locus to the 3.79–97.12 Mb region on the short arm of 4Ag (i.e., *Th. ponticum* chromosome LG34). Previous studies indicated that *Th. elongatum*, *Th. bessarabicum*, and *Pseudoroegneria* were probably the donor species of *Th. ponticum* (Muramatsu, 1990; Wang et al., 1991; Zhang et al., 1996a, Zhang et al., 1996b; Liu et al., 2018b; Yang et al., 2021). In the present study, the PCR products for 31 specific markers were sequenced and compared with *Th. ponticum* chromosome LG34 and *Th. elongatum* chromosome 4E. Eight of these PCR products were similar to chromosome 4E (sequence identities exceeding 85%) and reflected the relatively high synteny between chromosomes LG34 and 4E. These results further suggest that *Th. elongatum* is one of the donor species of *Th. ponticum*.

Powdery mildew is a devastating disease that can occur at all wheat growth stages. Genes conferring ASR and APR have been identified in wheat and its relatives. In general, the ASR-related genes are effective throughout the growth period and provide race-specific resistance, whereas the APR-related genes provide durable resistance at the adult plant stage and partial resistance to multiple pathogens (Roberts and Caldwell, 1970; Lin and Chen, 2007; Bai et al., 2022). Unfortunately, because the APR-related genes are very difficult to select on the basis of phenotypes, they have not been thoroughly characterized (Ren et al., 2012). The identified *Pm* genes conferring ASR (e.g., *Pm1a* and *Pm5e*) are easily overcome if the corresponding avirulence genes are mutated (Ren et al., 2017; Xie et al., 2020; Hewitt et al., 2021). The APR-related genes, which are considered to be more durable than the ASR-related genes, often encode allele-specific proteins that differ from NLR proteins (Bai et al., 2022). For example, *Pm38/Lr34/Yr18/Sr57* encodes ATP-binding cassette transporter (Krattinger et al., 2009), whereas *Pm46/Lr67/Yr46/Sr55* encodes hexose transporter (Moore et al., 2015). Therefore, to protect wheat plants from new *Bgt* strains, new *Pm* genes conferring APR will need to be identified and functionally characterized and ASR-related genes should be cloned and pyramided.

Among the *Th. ponticum*-derived *Pm* loci, only *Pm51* was confirmed to confer ASR and mapped to wheat chromosome 2BL (Zhan et al., 2014). Additionally, *Pm51* was derived from the wheat–*Th. ponticum* introgression line CH7086, which was obtained by crossing common wheat with the partial amphiploid Xiaoyan 7430. If CH7086 contains a compensating translocation, *Pm51* may have originated from the group 2 chromosome of *Th. ponticum*. Furthermore, the mc-FISH analysis indicated the six pairs of alien chromosomes in Xiaoyan 7430 do not include chromosome 4Ag (Jia et al., 2022). Therefore, we concluded that the powdery mildew resistance locus on chromosome 4Ag differs from *Pm51*. Similarly, the combined analysis of the compensating translocation and the PCR products for specific markers suggests the *Pm* genes conferring ASR and APR from the wheat–*Th. ponticum* substitution lines CH10A5 and SN19647 were derived from the group 1 chromosomes (Wang et al., 2020b; Li et al., 2021). However, the *Pm* gene conferring ASR and APR from the wheat–*Th. ponticum* translocation line 11-20-1 originated from a group 5 chromosome (Li et al., 2017). The *Pm* gene conferring ASR from the wheat–*Th. ponticum* translocation line WTT80 was also derived from a group 1 chromosome (Yang et al., 2022). According to the results of the Wheat 660K SNP array analysis and resequencing data, the adult-stage powdery mildew resistance-related gene of SN0293-2 and SN0293-7 may have come from a group 6 chromosome (Li et al., 2022). In the present study, the newly mapped APR-conferring *Pm* gene was revealed as the only *Pm* gene originating from a group 4 chromosome of *Th. ponticum*. This gene was derived from the wheat–*Th. ponticum* substitution line Blue 58, which was developed in 1976 (Mu et al., 1986). For over 40 years, this *Pm* gene has provided excellent resistance at the adult plant stage. Thus, it should be further explored and applied for breeding novel disease-resistant wheat lines. In addition, to exploit this APR-related *Pm* gene, the small alien segment in WTT146 has been transferred to the high-yielding variety Jimai 22 to obtain the novel and stable translocation line J146. The evaluation of disease resistance showed that J146 is highly resistant to powdery mildew at the adult plant stage. Notably, the assessment of agronomic traits indicated that YPP was not significantly different between J146 and its recurrent parent Jimai 22. The development of J146 may further promote the utility of this APR locus.

In summary, we developed five translocation lines carrying diverse alien segments by irradiating Blue 58 pollen. Of these lines, WTT139, WTT146, WTT323, and J146 were resistant to powdery mildew at the adult plant stage. By combining the results of our resistance evaluation, specific marker amplification, and alignment analysis, the *Pm* locus conferring APR was mapped to the 3.79–97.12 Mb region on the short arm of chromosome 4Ag. The identification of this powdery mildew resistance locus and its transfer into wheat cultivars by breeders may lead to the generation of novel disease-resistant varieties suitable for commercial cultivation.

## Data availability statement

The original contributions presented in the study are included in the article/**Supplementary Material**. Further inquiries can be directed to the corresponding author.

## Ethics statement

The authors declare that the experiments comply with the current laws of the country in which they were performed.

## Author contributions

ZL and QZ conceived the research; GY performed the experiments; GY and QZ drafted the manuscript. PD and WJ performed the sequence alignment using the genome of *Th. ponticum*. SF conducted adult plant stage resistance evaluation in Sichuan. HL and BL provided substantial help in preparing materials. All authors read and approved the final manuscript.
